# Chronic Open Infective Lateral Malleolus Bursitis Management Using Local Rotational Flap

**DOI:** 10.1155/2017/2728092

**Published:** 2017-09-17

**Authors:** Yong-Beom Lee, Dae-Hwan Kim, Jong-Ho Jung, Jae-Yong Park

**Affiliations:** Department of Orthopaedic Surgery, Hallym University Sacred Heart Hospital, College of Medicine, Hallym University, Anyang, Republic of Korea

## Abstract

**Background:**

Using a sinus tarsi rotational flap is an uncommon approach to treating chronic open infective lateral malleolus bursitis.

**Methods:**

We treated eight patients, including six males, using this approach. First, we debrided all the infected tissues and used a negative pressure wound closure system where needed. After acute infection had been controlled, the local rotational flap was used for cases where the wound could not be closed by a simple suture or bone exposure. The rotational flap was detached with a curved skin incision at the sinus tarsi next to the open wound and sutured to the defect, paying careful attention to the superficial peroneal nerve. The donor site was managed with a split-thickness skin graft.

**Results:**

The patients' mean age was 74.1 years. Six patients had a wound after suppurative infection, but two patients had ulcer-type bursitis. Six patients demonstrated full flap healing, but two patients had venous congestion necrosis.

**Conclusion:**

A sinus tarsi rotational flap is a useful method to ensure healing and coverage of chronic open lateral malleolus bursitis, especially for small to medium wounds with cavity and bone exposure.

## 1. Introduction

Bursitis is a common disease entity and could develop in every area of the body. The bursa tissue is a fluid-containing capsule lined with synovial cells. It can be divided into the anatomical bursa and the adventitious bursa. An adventitious bursa is created by abnormal shear force and is usually located in the subcutaneous tissue [1] and could develop in the lateral malleolus area because of the repetitive irritation, injury, and inflammation. Patients usually complain about pain, irritation, and discomfort [2]. The symptoms are not severe unless an infection is present, and thus conservative management, such as aspiration, compression, and injection, is the first-line treatment. However, the recurrence rate is high [1, 3], and infection may occur during treatment. When infection develops, bone may become exposed because of the thin overlying soft tissue. In some cases, the wound does not heal easily, particularly when patients have sensory loss or vascular impairment. When a simple closure is not possible, a skin graft, pedicled tissue transfer, and free tissue transfer can be used for covering the exposed bone. Each method has pros and cons, and some methods cannot be applied to certain patients because of his/her condition. We here describe the use of a sinus tarsi rotational flap as an uncommon approach to treating chronic open infective lateral malleolus bursitis and report the outcomes.

## 2. Materials and Methods

Institutional review board approval was obtained. From August 2012 to February 2015, we performed sinus tarsi rotational flap closure as a treatment for chronic open infective lateral malleolus bursitis in eight patients. As the first-line treatment, we attempted infection control surgery and primary closure. When closure was not possible or failed, we performed sinus tarsi rotational flap closure. Six of these patients were male, and five patients had the lesion on the left side. The average age was 74.1 (range: 52–88) years. We retrospectively reviewed the patients' chart, including medical history, wound duration, wound cause, pre-flap management, wound size, culture, surgery time (minutes), healing time (days), follow-up time (months), last wound status, and surgical complications. We categorized the wound size as small, medium, and large. When the defect only occurred in the lateral projection of the lateral malleolus and the diameter was less than 3 cm, the size was categorized as small. When the diameter was 3–5 cm, the size was considered medium. When the diameter exceeded 5 cm (i.e., a “large” wound), we did not perform this surgery.

### 2.1. Surgical Methods

**Figure 1:**
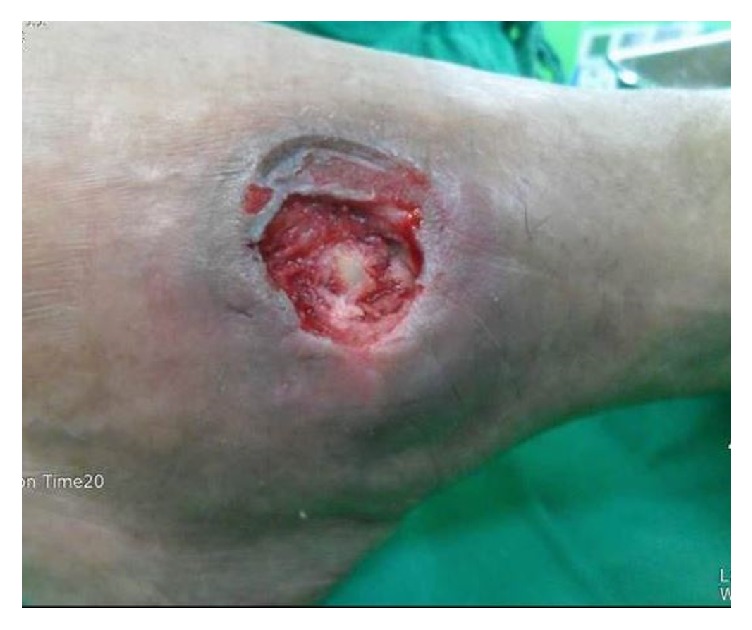
It is the lateral side of left ankle. The open wound has bony exposure. The infection is controlled state.

**Figure 2:**
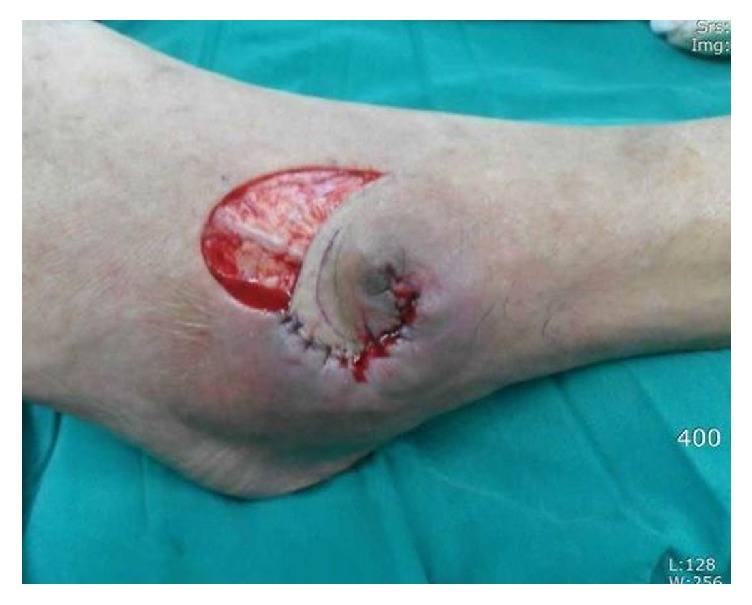
The detached flap was sutured to open wound end. The superficial peroneal nerve crosses the donor site.

All surgery was carried after infection had been controlled. Under general or spinal anesthesia, we used a sterilized thigh tourniquet at 300 mmHg for application of a split-thickness skin graft (STSG) from the ipsilateral thigh. The rotational flap was detached with a curved skin incision at the sinus tarsi beside the open wound and was sutured to the defect, with careful attention paid to the superficial peroneal nerve (Figures 1 and 2). The donor site was covered with the STSG immediately, and a tie-over dressing was applied for 5 days after surgery.

## 3. Results

**Figure 3:**
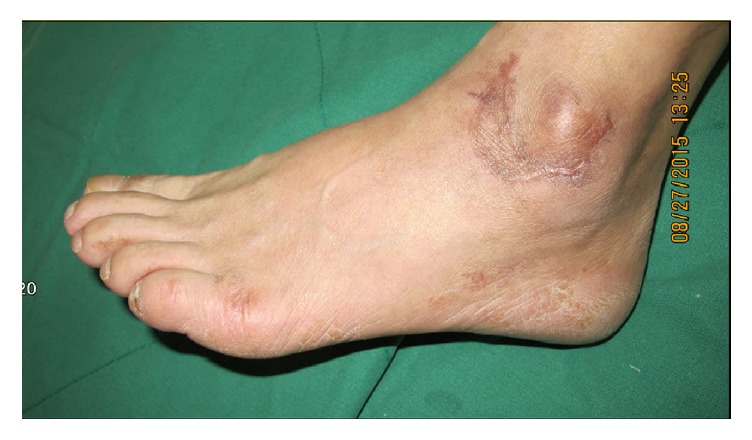
It shows the lateral side healed wound state. It was 6 months after surgery, number 4 patient.

**Figure 4:**
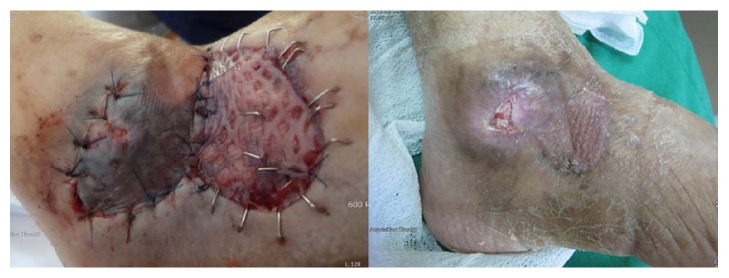
It was medium sized wound. The congestion developed after surgery. Finally the wound healed by scarring (number 2 patient).

**Figure 5:**
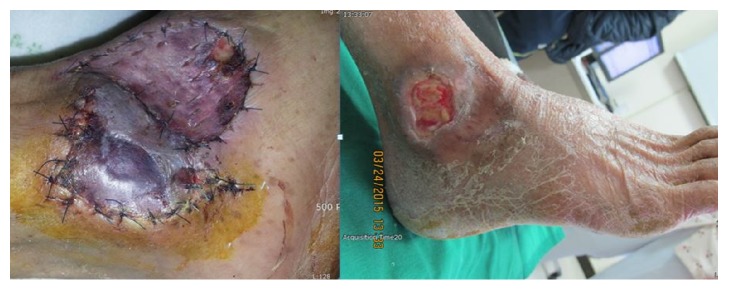
It was medium sized wound. The congestion developed after surgery. Finally the wound is not healed, and patient follow-up was lost (number 3 patient).

As elderly patients, all patients had several medical histories. Seven patients had hypertension, four patients had diabetes mellitus, and two patients had a history of cerebrovascular accident. Wound duration varied from 3 weeks to several years. In terms of the cause of the open wound, six patients developed the wound after suppurative infection (one was after a bursa resection, one was after a callus resection, and the others had spontaneous bursa infection). The remaining two patients had ulcer-type bursitis (one was due to being bedridden after pneumonia; one was due to pressure of a long duration). For the pre-flap management, three patients had only infection control surgery (debridement), and five patients underwent simple closure one to three times. In terms of wound size, three wounds were of a medium size, and five had small wounds. Upon tissue culture, various types of bacteria were found in each patient. The flap healed well in six patients (Figure 3), but venous congestion was seen in two cases after surgery. One was healed by scarring (Figure 4), but the other one did not heal (Figure 5). The surgery time, healing time, follow-up time, and other details are described in Table 1.

## 4. Discussion

The soft tissue around the ankle is thin. Moreover, the circulation of the leg may be decreased due to ageing and arterial insufficiency. These features make wound healing around the ankle problematic [4]. The area around the lateral malleolus is weaker because high pressure could be applied. This high pressure is usually generated by a regional specific cross-legged position or general weakness induced by external rotation of the hip. Adventitial bursitis could develop in this area. This type of bursitis does not have serious symptoms, but recurrence is common [1, 5]. With repeated recurrence, the possibility of infection is increased. There have been several reports about methods such as endoscopic bursectomy and sclerotherapy to address bursitis without recurrence [2, 3]. However, these methods are not suitable when an open wound has developed.

When an infective open wound develops in the lateral malleolus area, debridement is required for infection control. Sometimes, the bone is exposed during debridement, and dead space after simple closure limits wound healing. When a surgeon then attempts STSG, granulation continues for a long time before the skin graft begins. Moreover, the thin displaced skin tends to be broken easily. A pedicled tissue transfer, such as a sural flap, is usually used for a large wound [6]. However, this technique requires the patient to remain in the prone position, which is uncomfortable to older patients. Moreover, when the wound is small, the technique involved in using a sural flap is more difficult. The free tissue transfer procedure is a technically demanding procedure and cannot be used when a patient has a vascular disorder. Even if the tissue transfer is successful, the bulkiness of the soft tissue makes it difficult to find fitting shoes.

The rotational flap used in this study was simple and allows patients to be in a supine position. The donor site morbidity is reduced, because the skin next to the lateral malleolus (sinus tarsi area) is redundant and can be used for a full-thickness skin graft. Some contracture was found at the donor site, but this was released over time. Additionally, the tissue is not so thick as to complicate shoe fitting and is not so thin as to be broken easily. However, this rotational flap is not suitable for a large wound, because the rotational limb should be two to four times the size of the defect limb [7]. Additionally, if the wound is ulcerous, the donor skin is also too weak to cover the defect, slowing the healing.

In conclusion, use of a sinus tarsi rotational flap for an open lateral malleolus wound is a simple, effective, and rapid-healing approach for treatment of small to moderate sized wounds (≤5 cm), other than ulcerous wounds.

## Figures and Tables

**Table 1:** Detailed database of patients.

Number	Sex	Age	Wound duration	Cause	Past medical history	Pre-flap management	Wound size	Culture	Op time (min)	Anesthesia	Healing time (day)	Follow-up (months)	Last status
(1)	M	74	3 months	Ulcer (bedridden)	Old CVA, DM, HTN, Parkinson disease, congestive heart failure	Simple closure (×1) VAC application	Small (2 × 2 cm)	*E. coli*	60	General	43	8	Pod 8 mo ulcer recur
(2)	M	87	2 years	Ulcer (prolonged pressure)	DM, HTN	Simple closure (×1)	Medium (4 × 4 cm)	No growth	55	General	81	39	Venous congestion, scar healing, no problem
(3)	M	82	2 months	Infection	HTN, CKD	Debridement (×1)	Medium (5 × 5 cm)	*Morganella morganii*	50	Spinal	Not healed	4	Venous congestion, flap failure, follow-up loss
(4)	M	52	3 weeks	Infection	DM, HTN	Debridement (×1)	Small (2 × 2 cm)	*Streptococcus pyogenes*	50	Spinal	46	20	No problem
(5)	M	61	3 months	Infection after surgery	Gastric cancer	Debridement (×1)	Medium (3 × 3 cm)	MSSA	40	Spinal	14	25	No problem
(6)	M	87	Several years	Infection	Alcoholic dementia, HTN, DM, gastric cancer, adrenal insufficiency	Simple closure (×3)	Small (2 × 3 cm)	*Bacillus* species, MSSA	80	General	18	14	Pod 14 mo infection recur
(7)	F	88	4 months	Infection	HTN nephrectomy	Simple closure (×3)	Small (2 × 3 cm)	MRSA	85	Spinal	22	2	Well healed follow-up loss
(8)	F	62	4 months	Infection (after self-callus resection)	HTN, CVA, ITP	Simple closure (×2)	Small (2 × 3 cm)	*Pseudomonas*	90	General	16	20	No problem

Op: operation, CVA: cerebral vascular accident, DM: diabetes mellitus, HTN: hypertension, CKD: chronic renal disease, Pod: postoperative day; MSSA: methicillin sensitive *Staphylococcus aureus*, and MRSA: methicillin resistant *Staphylococcus aureus.*
